# Heavy Metals in Particulate Matter—Trends and Impacts on Environment

**DOI:** 10.3390/molecules30071455

**Published:** 2025-03-25

**Authors:** Ecaterina Matei, Maria Râpă, Ileana Mariana Mateș, Anca-Florentina Popescu, Alexandra Bădiceanu, Alexandru Ioan Balint, Cristina Ileana Covaliu-Mierlă

**Affiliations:** 1 Department of Metallic Material Processing and Environment Engineering, Faculty of Materials Science and Engineering, National University of Science and Technology Politehnica Bucharest, 313 Splaiul Independentei, 060042 Bucharest, Romania; ecaterina.matei@upb.ro; 2 Central Military Emergency University Hospital “Dr. Carol Davila”, 88 Vulcănescu, 010825 Bucharest, Romania; 3 Biotechnical Systems Engineering Doctoral School, National University of Science and Technology Politehnica Bucharest, 313 Splaiul Independentei, 060042 Bucharest, Romania; ancaflorentinapopescu@gmail.com (A.-F.P.); alexandra.hodoroaba@yahoo.com (A.B.); alexandruioan.balint@gmail.com (A.I.B.); 4 Faculty of Biotechnical Systems Engineering, National University of Science and Technology Politehnica Bucharest, 313 Splaiul Independentei, 060042 Bucharest, Romania; cristina_covaliu@yahoo.com

**Keywords:** heavy metals, particulate matter, environmental pollution, health risk, biomonitoring

## Abstract

Heavy metals represent a class of pollutants detected at concentrations lower than 10 ppm in different matrices that are intensively monitored due to having a major impact on human health. Industrial activities including mining, agriculture, and transport, determine their presence in different environments. Corrosion phenomena of various installations, volcanic eruptions, or atmospheric deposition on the soil surface and in water can contaminate the respective environments. Atmospheric pollutants in the form of suspended dust particles with diameters below 10 microns are predominantly composed of different metallic species from Cd, Cr, Cu, Ni, etc. This paper presents a review of the main sources and types of heavy metals present in the atmosphere in the composition of particulate matter (PM), highlighting the main mechanisms of occurrence and detection techniques, including the impact on bio-geo-chemical processes in the soil and food chain, in close correlation with their impact on environment and human health. The purpose of this review is to highlight the current level of knowledge regarding the global situation of heavy metals in PM and to identify gaps as targets for future research.

## 1. Introduction

Air pollutant concentrations have increased considerably over the last decade. The most common sources of air pollution are motor vehicles, the production of industrial goods and the consumption of raw materials, household combustion devices, and forest fires [1,2]. Common air pollutants are particulate matter (PM_10_ and PM_2.5_), ozone (O_3_), nitrogen dioxide (NO_2_), carbon monoxide (CO), and sulfur dioxide (SO_2_), but also others such as heavy metals, black carbon, or persistent organic pollutants. These pollutants are responsible for health problems such as asthma, heart disease, and stroke. In 2022, air pollution in the EU-27 Member States was estimated to cause approximately 239,000 annual deaths from exposure to fine particulate matter (PM_2.5_), 70,000 from ozone (O_3_), and 48,000 from nitrogen dioxide (NO_2_) [3]. PM contains microscopic solids or liquid droplets that pose a risk of inhalation, which can lead to serious health problems. Those with a diameter of less than 10 μm (PM_10_) can penetrate deep into the lungs, as some studies indicate that they may even reach the bloodstream. Those with a diameter less than 2.5 μm (PM_2.5_) are the ones that pose the greatest health risk [4]. PM, due to having small diameters, can remain suspended in the atmosphere for long periods and travel long distances from the emission source, thus posing a threat to large geographical areas. The chemical composition of PM varies depending on the source of generation, from nitrates, sulfates, elemental and organic carbon, organic compounds (e.g., polycyclic aromatic hydrocarbons), biological compounds, and metals. Characterization and determination of the chemical composition of PM are essential for pollution reduction. Over time, PM can also deposit on soil surfaces, waters or higher plants, roots, and leaves, leading to adverse effects on ecosystems and affecting their biodiversity [5].

In urban environments, the occurrence of heavy metals (HMs) can stem from various sources such as car workshops, combustion, or tire wear. These can be transferred from one environmental compartment to another in the biosphere, through long-range transboundary air pollution. Also, their persistence in ecosystems could lead to bioaccumulation in the food chain [6]. Plants filter the air by adsorbing PM and implicitly HMs, as evidenced by the decrease in PM concentration in forests [7,8]. Thus, depending on the characteristics of the leaves, plants can contribute to the retention of PM and HMs and air filtration. The deposition of HMs from the atmosphere into marine waters contributes to their presence in marine organisms. In Europe, between 2010 and 2021, nine hazardous substances were monitored in marine organisms, including Cd, Pb, and Hg, which exceeded the limit values stated to protect human health, according to a report by the European Environment Agency (EEA) published in 2024 [9]. Thus, about 49% of surface waters are contaminated with Hg. Atmospheric deposition of metals has also contributed to their exceeding concentrations in agricultural soils, with Pb and Cd levels exceeding the limits by 47% and 31%, respectively, according to a report by the European Topic Centre on Data Integration and Digitalization (ETC DI) [9].

Also, HMs are natural elements that can be found in the Earth’s crust. The main source of environmental and human contamination comes from anthropogenic activities specific to any activity that contains metals and their compounds [10,11,12]. In addition, environmental contamination can also occur through metal corrosion, atmospheric deposition, soil erosion and leaching, and evaporation of metals from water resources in the soil and groundwater [12]. Natural sources of contamination including HMs are phenomena such as weathering and volcanic eruptions. Some metals such as (Co), copper (Cu), chromium (Cr), iron (Fe), magnesium (Mg), manganese (Mn), molybdenum (Mo), nickel (Ni), selenium (Se), and zinc (Zn) are essential nutrients required for various biochemical and physiological functions, and their lack leads to deficiencies, diseases, or syndromes [12,13].

Metals such as Al, Sb, As, Ba, Be, Bi, Cd, Ga, Ge, Au, In, Pb, Li, Hg, Ni, Pt, Ag, Sr, Te, Tl, Sn, Ti, V, and U have no established biological functions and are considered non-essential for plants [14]. The literature indicates that the weight and toxicity of HMs are interdependent, which is why the category also includes arsenic (As), a metalloid that possesses toxicity even at low levels of exposure. As levels in the air vary from 1 to 3 ng/m^3^ in isolated areas (far from human emissions) and from 20 to 100 ng/m^3^ in urban areas [15,16]. HMs found in biological systems affect cell organelles, cell membranes, mitochondria, lysosomes, the endoplasmic reticulum, nuclei, and some enzymes involved in metabolism, detoxification, and damage repair [12].

Because HMs are also nutrients or trace elements, it is important to study their bioavailability at concentrations below 10 ppm in different environmental compartments. Their bioavailability is influenced by physical factors from temperature to sequestration and adsorption processes, chemical factors such as liposolubility, kinetics and complexation processes, and biological factors such as species characteristics. HMs are part of enzymes and participate in redox reactions, exercising key roles in biochemical processes in plants and animals. To the same extent, HMs can generate toxicity and carcinogenicity, the mechanisms being however not clearly elucidated or understood. As a general rule, each metal presents unique physicochemical characteristics and properties that confer toxicity, such that As, Cd, Cr, Pb, and Hg, which are priority metals for public health. The production of reactive oxygen species (ROS) and oxidative stress play a key role in the toxicity and carcinogenicity of these metals [17,18,19,20,21]. HMs interact with DNA and nuclear proteins, causing damage and conformational changes that can lead to carcinogenesis or apoptosis [22].

The accumulation of HMs through atmospheric deposition can lead to their buildup not only in soil and water but also in crops or plants, causing damage and alterations to physiological functions in animals or humans through the food chain [23]. For example, Cu is essential for enzymes related to oxidative stress, being an essential nutrient in plants and animals. Therefore, it is part of the class of metalloenzymes responsible for hemoglobin formation, carbohydrate metabolism, catecholamine biosynthesis, and the cross-linking of collagen, elastin, and hair keratin. It has two oxidation states and participates in redox reactions [10,24]. Its toxicity is due to the transitions between Cu(II) and Cu(I) that can lead to the formation of superoxide and hydroxyl free radicals, and repeated exposure to Cu leads to cellular damage and eventually to Wilson’s disease [16,23,25,26]. Plants used as bioindicators of pollution can be sensitive species, which suffer structural and morphological damage when exposed to pollutants, and accumulator species, within which pollutants accumulate. There are also species that become abundant and proliferate in heavily polluted areas. The way in which plants such as lichens and large plants respond to air pollution can lead to the possibility of early detection of impaired quality [24,27].

In this context, the purpose of this review is to highlight the current level of knowledge regarding the global situation of heavy metals in PM and to identify their sources, monitoring processes, health issues, and gaps which can serve as targets for future research.

## 2. Characteristics of Heavy Metals in Particulate Matter from Air

### 2.1. Classification of Particulate Matter

**Figure 1:**
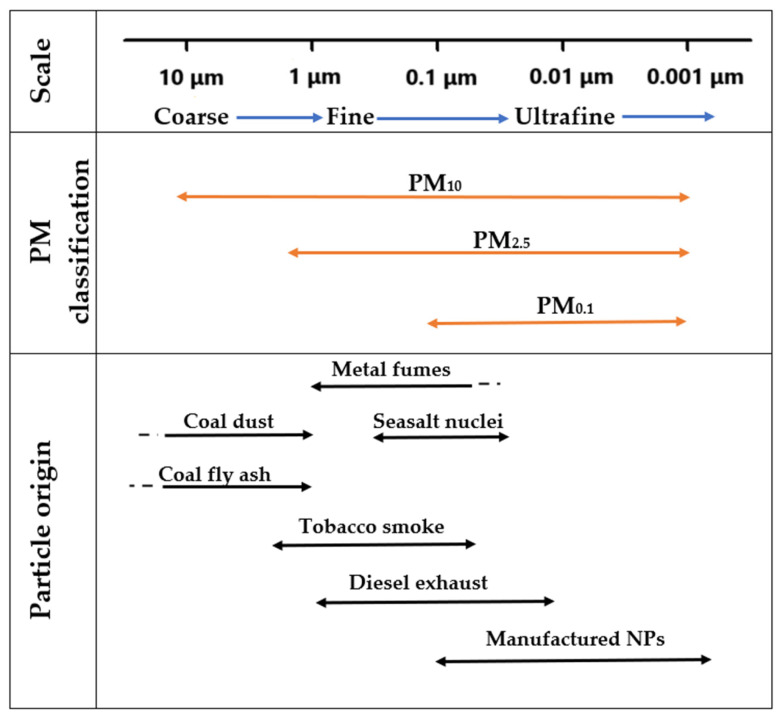
Classification of particulate matter (PM) and sources of particles. Adapted from [31].

Particulate matter (PM) comprises solids and/or liquids dispersed in the atmosphere, displaying differences in size, form, and chemical properties [28]. It is categorized based on size as follows: PM_10_, coarse particles with an aerodynamic diameter ≤10 μm; PM_2.5_, fine particles with an aerodynamic diameter ≤2.5 μm; and PM_0.1_, ultrafine particles with an aerodynamic diameter ≤0.1 μm [29]. The latter are studied from the point of view of nanotoxicology and occur both naturally and from combustion processes or from cosmetics, medicines, and catalysts [28,30]. The formation of PM is based on chemical reactions between contaminants in the environment, their size, and their origins, as represented in Figure 1.

In air, the most frequent form of HMs is PM_2.5_ or PM_10_. PM can be emitted directly into the atmosphere as primary particles from combustion, dust, and soot, while others are formed in the atmosphere as secondary particles resulting from emissions of SO_2_, NO_X_, and NH_3_.

Primary PM, such as black carbon (BC), salt aerosols, and mineral dust, is relatively stable compared to secondary PM, but the tendency to mix with other air components and hygroscopicity can cause aging processes during transport [32]. During transport, through the presence of other pollutants, the risks to human health become higher.

PM can also interact with other atmospheric components and can be associated with the formation of secondary pollutants, such as ground-level ozone and harmful gases [5,33]. Secondary PM, with NO_X_ as a precursor, can produce HNO_3_ in the form of aerosols and nitrate particles through oxidation. SO_2_ is also oxidized to sulfate in the form of particles, but to a lesser extent than NO_x_ and leads to the occurrence of sulfate particles. In addition, volatile organic compounds (VOCs) can react with oxidants in the form of OH, NO_3_, and O_3_ to generate oxygenated VOCs and subsequently secondary organic aerosols (SOAs) [34,35].

### 2.2. Air Quality Assessment

In the composition of PM originating from industrial areas where metals are commonly used or in areas with intense traffic, HMs appear frequently. The most commonly found metals are those that are also found in the Earth’s crust (Fe, Al, Cr), produced by traffic and catalysts (Sb, Cu, Pt, Pd, Ru), from fertilizers (Cu, As), and other metals such as Pb, Cd, Co, Mo, Ni, Hg, V, and Zn [30].

**Figure 2:**
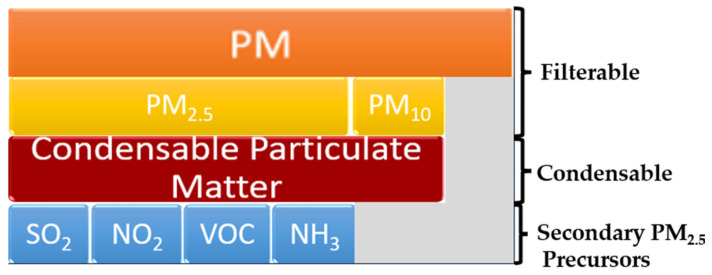
Filterable, condensable, and secondary PM_2.5_ precursors. Adapted from [36].

The National Ambient Air Quality Standard (NAAQS) from the USA, in accordance with greenhouse gas (GHG) emissions and monitoring, divides PM into three subgroups, PM, PM_10_, and PM_2.5_, which are defined as shown in Figure 2 [36]. PM is dust of any size (usually less than 30 microns in diameter) that is separated by a filter. PM_10_ represents filterable particles and condensable particles, i.e., gaseous emissions that condense and form suspended particles at ambient temperature. They pass through a filter positioned at the exit of a stack. PM_2.5_ comprises condensable and secondary particles. The latter are those that are formed in the atmosphere after pollutants from fuel combustion leave the stack. In the presence of sunlight and water vapor, some of the SO_2_, NOx, VOCs, and ammonia (NH_3_) react chemically and lead to the formation of PM_2.5_. This could be reduced by precursor reducing.

When PM is present indoors, for example, at schools, people are affected. The indoor environment influences the presence of PM and its concentrations, from the type and age of the room, height, ventilation, etc. It has been found that students in Europe and Asia are more exposed to PM in classrooms, compared to other regions, when the limits recommended by the World Health Organization (WHO) are exceeded [37].

PM_10_ and PM_2.5_ concentrations vary from country to country, region to region, and even within the same space. Expressed as average concentrations in classrooms, in Europe, PM_2.5_ could reach up to 100 μg/m^3^ and PM_10_ could reach 140 μg/m^3^; in Asia, PM_2.5_ and PM_10_ could be recorded up to 90 μg/m^3^ and 284 μg/m^3^, respectively; in North America, levels could only reach 16 μg/m^3^ for PM_2.5_ and 23 μg/m^3^ for PM_10_; and in South America, levels could reach 15 μg/m^3^ and 20 μg/m^3^ for PM_2.5_ and PM_10_, respectively [37]. In recent years, a new category of PM has started to be monitored with particles less than 100 nm, known as PM_0.1_.

Indoor air pollution and pollutants can be caused by inadequate ventilation systems, poor cleanliness, and high occupancy rates. The use of filters can reduce PM by up to 50%, in addition to addressing structural or heating and cooling issues in buildings.

Air quality assessment is closely linked to the connections between O_3_ and PM, which are key pollutants causing cardiovascular and respiratory problems [38,39]. The presence of these two pollutants influences radiative forcing, leading to climate change and the greenhouse effect [40,41]. Because they have relatively long lifetimes, they can be transported over long distances, even thousands of kilometers from the source of generation, through cross-border transport [34].

The decline in PM_2.5_ and PM_10_ levels, combined with the recent challenges posed by COVID-19, has been associated with increased mortality. Current studies are exploring a causal relationship between pollution, especially with PM_2.5_, and the severity of COVID-19 infections [42]. PM_2.5_ affects human health in urban areas, reaching the lungs and alveolar regions, whereby PM_0.1_ can also reach the blood [43].

Total particulate matter (TPM) is composed of filterable particulate matter (FPM) and condensable particulate matter (CPM). FPM is well monitored, while methods for CPM monitoring are still under investigation. Epidemiological studies have shown that morbidity and mortality resulting from respiratory diseases are correlated with exposure to PM_2.5_ [44]. These CPM particles have very small diameters and large specific surfaces, attracting toxic and harmful substances, such as HMs and viruses [45].

CPM includes unconventional pollutants which are not included in standards from different countries. SO_4_^2–^, NO_3_^–^, and Cl^–^ ions may appear in CPM from the transformation of gaseous pollutants such as SO_x_, NO_x_, and HCl existing in flue gases [46,47,48]. NH_4_^+^ ions appear from the selective catalytic reduction of NH_3_ or wet flue gas desulfurization [49,50]. Organic components such as PAHs and VOCs in CPM come from the volatilization and incomplete combustion of fuel, along with ketone compounds, alcohols, and esters [51,52]. Metals come from the vaporization of elements and their tendency to volatilize, resulting from the combustion of fuels, incinerators, and fine particles [47,48,51].

### 2.3. Analytical Detection of Heavy Metals and Particulate Matter

#### 2.3.1. Preparation of Samples for HM and PM Detection

The mineralization of aqueous samples containing heavy metals is essential for removing organic matter. The determination of Cr, Se, Zn, Cd, Co, Cu, Mo, Ni, Pb, Hg, and As in soil samples can be achieved using the atomic absorption spectrometry method with flame atomization (AAS FM) after soil mineralization with a mixture of concentrated HNO_3_ and HClO_4_ in a 3:1 ratio [53]. The mineralization technique is the most commonly used method for preparing samples containing HMs. It usually involves the use of concentrated mixtures of chemical reagents and the elimination of volatile components. For instance, the preparation of samples for metal analysis in stationary source emission samples collected on quartz filters includes acid digestion using fluoroboric acid (HBF_4_) and HNO_3_ solutions, as well as acid extraction in an *aqua regia* solution (a molar ratio of HNO_3_ to HCl of 1:3) as an alternative to the standard digestion method with HF [54]. Using this technique, the quantitative recovery of CRM and LOD is comparative or better than that achieved with the HF technique. A disadvantage of this technique is the formation of salts, which can lead to increased signal drift and potential interferences.

Concentrations of Mn, Pb, Ni, V, Cd, and Co in PM_10_ and PM_2.5_ were analyzed using the microwave digestion inductively coupled plasma mass spectrometry (ICP-MS) method [55]. The samples were collected with a medium-flow sampler equipped with PM_2.5_ and PM_10_ cutters, operating at a flow rate of 100 L/min and a height of 1.5 m. The study showed that the concentrations of Mn and Pb were relatively high in both PM_10_ and PM_2_.5. Additionally, the ratio of these HMs in PM_2.5_/PM_10_ often exceeded 0.5, suggesting a preference for HMs to concentrate in PM_2.5_.

Another study detailed the preparation of biomonitoring moss samples including *Philonotis marchica*, *Hylocomium splendens*, and *Homalothecium philippeanum* containing Cu, Fe, Mn, Ni, Pb, and Zn using microwave mineralization with a mixture of 67% HNO_3_, 30% H_2_O_2_, and 40% HF, analyzed via the ICP-OES method [56]. A significant drawback of microwave digestion for trace HMs is the potential release of gaseous oxidation products, which can lead to a sudden increase in pressure [56].

Additional techniques used for the mineralization of samples containing HMs include direct solid sampling methods, such as laser ablation and electrothermal vaporization (ETV), as well as emulsified systems, enzymatic digestion (utilizing proteases, amylases, and lipases), alkaline treatment, slurry sampling, and bioremediation approaches [57,58,59]. Kaur et al. [58] studied microbial-induced calcium carbonate precipitation (MICCP) for the bioremediation of Cd and As from artificially contaminated wastewater. Using atomic absorption spectroscopy (AAS), the authors observed removal efficiencies of 93.13% for Cd and 94.25% for As. The objective of employing these techniques is to enhance result quality, reduce the maintenance requirements of analytical equipment, and support the demand for environmentally sustainable procedures while ensuring the health and safety of personnel.

The Compendium of Methods for the Determination of Toxic Organic Compounds in Ambient Air-Second Edition, approved by the United States Environmental Protection Agency (U.S.EPA), is also used for sample collection and the determination of HM concentrations [60]. The concentrations of 22 HMs in ambient air samples collected around Bakhtegan Lake in Hajiabad village were analyzed using ICP-MS every three days during the summer of 2019 [60]. The authors used a PTFE filter characterized by a diameter of 47 mm and a pore size of 0.5 μm. Sampling was conducted at a flow rate of 8 L/min for 24 h. The results obtained showed an average PM_10_ concentration of 78.12 μg/m^3^ around the lake, exceeding the World Health Organization’s recommended limit of 50 μg/m^3^ for PM_10_. Among the HMs, Fe showed the highest concentration, while V had the lowest.

PM collection can also be conducted using equipment that separates PM into fractions [61]. Various types of specific filter and membrane materials are evaluated based on filtration efficiency, pressure drop, density, and porous structure [62]. Traditional filter material membranes such as cellulose acetate/nitrate, glass fiber, Teflon, or quartz (membrane) offer air pollutant removal efficiencies higher than 99% [55,63]. According to [61], the filter support should be made either of stainless steel, anodized aluminum, aluminum alloy, polycarbonate, POM, or PTFE grid material. However, the impact of filter sub-sampling on PM analysis should be investigated [64].

Some examples of advanced fiber filter materials used for PM separation from air are presented in Table 1.

Efficient filtration is achieved when the pores of the filter material are smaller than the particles to be separated, and in the case of nanofibers, they must be thicker. In both cases, a high resistance to air flow and a high pressure drop are required. There are also inertial filters that have led to the development of inertial impactors that operate at low flow rates and can collect aerosol particles, with the filtration channel being enlarged dozens of times compared to the average size of PM particles. The materials from which they can be manufactured are diverse, with a low cost, thermal resistance, and stability [71].

In the case of school classrooms, air samples have been collected using low-volume air samplers with a suction flow rate of 5 L/min, starting just before the beginning of classes and continuing until approximately 30 min after the class ended [56].

#### 2.3.2. Methods for HM and PM Detection

Energy-dispersive X-ray fluorescence spectrometry is another analytical technique used for the quantitative determination of Mn, Pb, Cu, As, Cr, and Ni contained in PM_2.5_ [72]. Other metal elements such as Al, Ca, Fe, Mg, Na, K, Cu, and Cd can be analyzed using inductively coupled plasma-mass spectrometry (ICP-MS) [73] or inductively coupled plasma-optical emission spectrometry (ICP-OES) [74] or qualitatively detected based on their energy spectra. Atomic absorption spectrophotometry (AAS) employing either the graphite furnace or flame technique can also be used for the quantitative determination of HMs in air, soil, or water matrices [75].

In recent years, advanced technologies such as artificial intelligence (AI) and machine learning (ML) have been integrated into physical, chemical, and biological methods for HM removal to enhance the efficiency and effectiveness of treatment processes [76]. The application of AI in HM detection and removal involves the use of advanced computational algorithms and models to identify, measure, and eliminate toxic metals such as Pb, Hg, Cd, As, and Cr from water matrices. AI facilitates complex data analysis, real-time monitoring, and optimization of water treatment processes, providing fast and accurate solutions to protect public health and the environment [76]. Machine learning (ML) in HM detection and removal involves applying ML algorithms and techniques to analyze, interpret, and manage metal contaminants in water matrices. These algorithms can process large volumes of data and identify complex patterns, providing accurate predictions of HM concentrations and optimizing treatment processes to maximize the removal efficiency of toxic contaminants such as Pb, Hg, Cd, As, and Cr [76].

The determination of the concentration of PM_10_ or PM_2.5_ in ambient air must comply with the maximum uncertainty limits prescribed by Directive 2008/50/EC and EN 14,385 [77,78]. To ensure accurate sampling, it is recommended to use two samplers of the same model consecutively or four samplers of the same model separately, operating at a nominal flow rate of 2.3 m^3^/h under ambient conditions. When using a single filter, the sampling period should follow a 24 h cycle in the laboratory, with a precision within 1 min. EN 14,385 [78] specifies that air concentration measurements should range from 1 μg/m^3^ to 150 μg/m^3^ for PM_10_ and up to 120 μg/m^3^ for PM_2.5_ during 24 h sampling periods.

Air filtration remains the most common technique for separating PM into fractions. PM sampling is performed following established EPA and EN ISO methods, whereby PM is extracted from a volume of air through filtration or impaction and centrifugation.

For PM from ambient sources, samplers such as cyclones and impactors are used. These methods follow the specific standards such 40 CFR part 50 of the EPA and EN 12341 of the EU [64,79].

The partition of specific PM fractions was carried out by Wang et al. [55] in Tianjin, China, by using the gravimetric method. Investigations conducted from 1 March to 30 May in 2021 revealed that the daily mass concentration of PM_10_ was 140 μg/m^3^, which was higher than that of PM_2.5_ (90 μg/m^3^). The ambient air quality standards in China are 150 μg/m^3^ for PM_10_ and 75 μg/m^3^ for PM_2.5_.

Studies on the impact of PM on the environment are also carried out by monitoring their morphology and composition in close connection with vegetation. Thus, tree leaves and plant parts (root, stem, leaves, needles) can be studied by using electron microscopy (SEM) and energy-dispersive X-ray (EDX) techniques [43]. Studies indicate that PM up to 2 mm can penetrate the stomatal cavity and the most furrowed areas of leaf surfaces. Song et al. [43] determined that about 96% of the deposited particles were smaller than 2.5 mm on tree leaf species, with species such as *Juniperus Formosan* being effective at attenuating PM on leaves.

A method for identifying sources generating PM and, consequently, HMs involves using isotopes as novel markers to track pollution sources in environmental, geochemical, and biological systems [26,80,81,82,83]. Stable isotope fingerprinting has been increasingly applied in environmental studies in recent years for processes such as the absorption of Hg and Cd in rice, the monitoring of Tl or V in soil and water, and the tracking of PM movement [82,84,85,86]. For PM, eight stable isotopes have been used so far in the form of metals and metalloids: Pb, Zn, Fe, Hg, Si, Cu, Sr, and Nd. The use of non-traditional stable isotopes for determining PM sources is currently limited to local sites. Improving isotopic analytical methods is important for advancing air pollution analysis and control, particularly through the development of accurate source apportionment methods.

Super Masscolloider (SMC) sheets, fabricated from refined nanocellulose fibers, were used for the first time as filters to capture PM_2.5_ and PM_10_ dust particles [87]. The results showed that the SMC sheets captured 230 mg of PM_2.5_ dust particles within 30 min, exceeding commercial-grade micro-glass fiber filters, which captured only 113 mg of PM_2.5_ dust particles in the same duration.

Unmanned Aerial Vehicle (UAV) technology is one of the emerging innovations that enables real-time vertical measurement of particulate matter concentrations and aids in acquiring high-resolution particulate matter profiles [88].

The Andersen Cascade Impactor, which complies with the threshold limit values (TLVs) set by the OSHA and USEPA, is also used for determining particle size. For particle size classes ranging from 0 to 10 mm collected during biomass combustion in the Amazon forest, the sampling calibration flow was 28.3 L/min [89].

Future trends in particulate matter (PM) monitoring emphasize advancements in sensor technology, focusing on the design and refinement of sensors specifically developed for detecting PM in urban environments [90].

#### 2.3.3. Quality Assurance/Quality Control (QA/QC) Procedures

Quality control is essential to ensure that the uncertainties in the measured values of PM and HMs in ambient air remain within the specified limits during extended field monitoring periods.

Temperature and pressure sensors in the sampler must be calibrated annually to ensure that the action criteria of ±3 K and ±1 kPa, respectively, are not exceeded [61].

For HM analysis, it is recommended to use appropriate standards (certified reference materials—CRMs) for a defined number of samples. The calibration curve should be established based on the CRM, and recovery should be regularly checked. The instrumental limits of detection (LODs), limits of quantification (LOQs), recovery rate for spiked samples, and relative standard deviation (RSD) must be determined for each metal analyzed. The coefficient of determination (R^2^) of the calibration curve should be ≥0.9980 to ensure high accuracy and linearity. Additionally, the reagents and chemicals used should be of analytical grade. Samples should be analyzed in triplicates to ensure that the variance does not exceed 5%.

According to EN 14385 [78], the measured concentrations for As, Cd, Cr, Co, Cu, Mn, Ni, Pb, Sb, Tl, and V should not deviate by more than 10% from the certified value or should fall within twice the uncertainty value specified for the certified reference materials (CRMs).

## 3. Sources of Heavy Metals and Particulate Matter in Atmosphere

### 3.1. Heavy Metals Sources

HMs can be transported from one environment to another in the biosphere, and they can be toxic to organisms even at low concentrations. They are emitted into the environment from a variety of anthropogenic sources and practically supplement the natural background of geochemical sources. The oldest cases of environmental pollution in the world were due to the extraction and use of heavy metals, for example, the mining of Cu, Hg, and Pb, and their smelting and use by the Romans [91].

**Figure 3:**
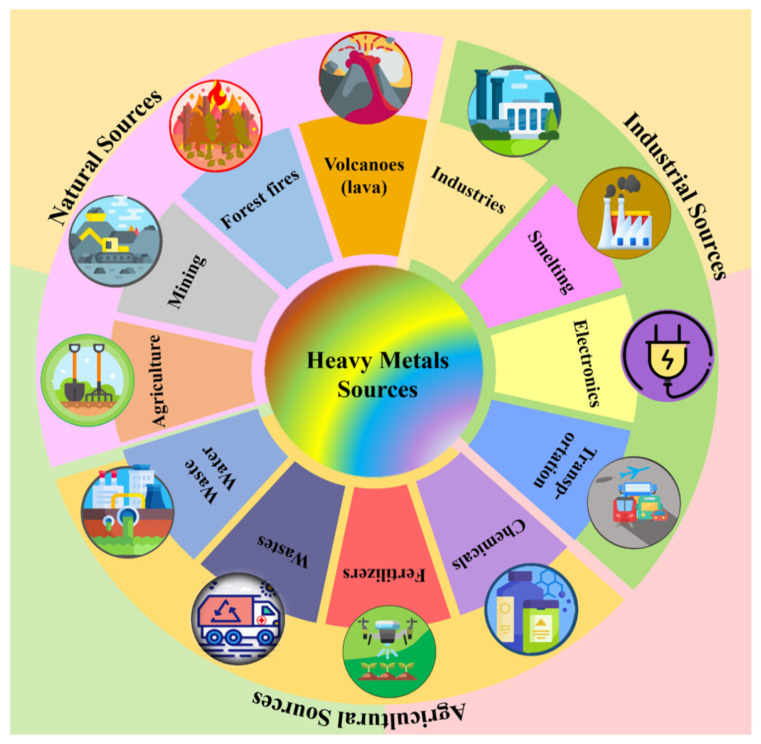
The various sources of heavy metal pollution. Reproduced from [92] with permission from Elsevier.

Anthropogenic sources of HMs lead to the occurrence of As, Hg, Cd, Cr, Ni, and Pb in the atmosphere, with the specification that there is a continuous concern to reduce these emissions in Europe over the last 40 years. The literature includes as sources of HM emission coal combustion in power plants, industrial, residential, and commercial boilers, oil combustion in various processes, iron and steel production, cement, non-ferrous metal manufacturing, waste incineration, and metal processing in various industrial activities [92]. One of the most comprehensive classifications regarding pollution sources is represented in Figure 3.

HMs are mainly found in the atmosphere and street dust, especially in urban areas, originating from industrial activities, cement production, traffic, building erosion, and fossil fuels. Street dust containing HMs becomes toxic due to the presence of As, Cr, and Pb, which are persistent and represent a major risk to human health [93]. Penetration into the body can occur through digestion, respiration, and the skin, leading to metabolic disorders, even poisoning, nervous system dysfunction, etc. HMs from the atmosphere can be transferred to surface waters, thus affecting the food chain and eventually being transferred to humans.

Incineration of municipal and industrial solid waste is one of the major sources of HMs in the atmosphere, including Pb, Cd, Zn, and Cu. These HMs can reach soil and surface waters or plants through deposition and runoff, transferring their potential pollutants and influencing the bioaccumulation capacity in other environments [94].

Continuous urbanization has led to an increase in the quantities and types of municipal solid waste. Among these, electronic waste is an important source of HMs, with risks for the environment. Waste incineration results in particulate matter and fly ash, both of which impact the environment. In addition, fly ash contains not only HMs but also organic pollutants (e.g., dioxins, furans, VOCs, and PAHs) [94].

Pacyna et al. reported that fuel combustion to produce heat and electricity generated As, Cd, Cr, Ni, and Pb in amounts of 391 t, 367 t, 1394 t, 3795 t, and 1623 t, respectively [1]. Gasoline combustion released 6772 t of Pb in atmosphere [1].

### 3.2. Particulate Matter Sources

**Figure 4:**
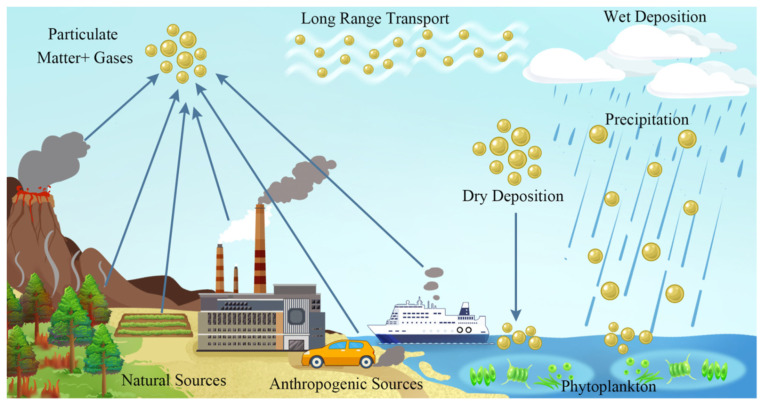
Schematic illustration of atmospheric emission sources and deposition processes. Reproduced from [97] with permission from Elsevier.

Referring to the presence of PM in atmosphere, the types of sources that contribute to its presence can include inorganic compounds, dust, sea salt, traffic, biomass combustion, and fuel combustion (Figure 4). Inorganic species are secondary sources of sulfate, nitrate, and mixed secondary inorganic aerosol (SIA), originating from the oxidation of SO_2_, which comes from the combustion of sulfur in fossil fuels and large combustion plants, power plants, and marine diesel engines, and NO_2_, originating from NO emitted by the high-temperature combustion of gas, liquid fuels, and coal. The oxidation of NO_2_ and SO_2_ occurs by hydroxyl radicals and in droplets present on the surfaces of particles [95,96]. Nitrate predominates in winter when temperatures are lower, favoring the formation of ammonium nitrate particles. Sulfate usually peaks in summer, when increased photochemical activity allows for a more homogeneous formation of sulfate from emitted SO_2_. SIA can also be formed through oxidation.

Dust, as a source of PM, can come from natural soil or deserts, traffic areas resulting from brake materials (Cu, Sb, Si, Fe), or tire wear (Zn). Sea salt contains a large amount of chloride, but as the particles age, this is replaced by sulfate and/or nitrate, so a mixture of particles makes up the source of PM. Emissions from motor vehicles and those from spark ignition (petrol) and compression ignition (diesel) are other sources of PM. Biomass used for residential heating/cooking, whether from wood or other sources (crop residues, manure, etc.), also leads to the occurrence of PM [95].

Primary PM originates from coal-fired boilers and consists of filterable particulate matter (FPM) and condensable particulate matter (CPM), which are among the main sources of airborne PM contributing significantly to PM_2.5_ levels. Their control is currently well established, with stack emissions being substantially reduced in coal-fired power plants. Studies on limiting CPM in the atmosphere refer to methods that include wet flue gas desulfurization and dust reduction. PM emissions from coal combustion are the main source of PM in flue gases [98].

The diversification of PM sources through increasing industrialization and intense urban development has induced a heterogeneous composition of PM, with variable sizes and different aggregation states, including sulfates, nitrates, ammonium, sodium chloride, carbon, metals, and water [99]. PM deposition on surfaces can occur in different ways—dry deposition by particles settling from the air or wet deposition via meteoric waters (rain or snow) and wind-driven cloud water (occult deposition by cloud, fog, etc.)—with the time of year influencing these types of deposition. Secondary PM emissions are about 1.7 times higher in the dry season compared to the rainy season [99,100].

PM from coal combustion contains toxic heavy metals such as Cd, Cr, Cu, Pb, Zn, and As. If the source is a domestic one resulting from biomass combustion, PM will mainly contain organic compounds, including polycyclic aromatic hydrocarbons (PAHs) [101].

There are also various tree species that can act as canopies for PM released into the atmosphere. Wildfires themselves can create PM and gaseous pollutants; so, although trees can protect against PM, burning releases them back into the atmosphere. A study conducted in the Metaponto Nature Reserve in southern Italy indicated that the species *Acacia saligna* (Labill.) *Wendl.* and *Pistacia lentiscus* L. were affected and unsafe due to releasing large amounts of PM during burning [102]. It was found that PM resulting from urban pollution and biomass burning accounted for about 30% of the total PM sources found in the Amazon [103,104].

## 4. Influence of HMs from Air in Other Environments

### 4.1. Water

Rainwater is an indicator of the presence of HMs in the atmosphere. An example is the monitoring of Pb isotopes in rainwater samples following the introduction of unleaded petrol, which led to a decrease in the Pb level in water. Rainwater specimens gathered from 25 sites across Scotland revealed a ^206^Pb/^207^Pb ratio of 1.126 ± 0.006 [26,105]. Measures to reduce the consumption of leaded petrol have led over time to a reduction in the ^206^Pb/^207^Pb isotopic ratio of rainwater in the UK [105].

Through atmospheric deposition, PM can reach aquatic ecosystems (lakes, rivers, oceans), influencing the concentration of essential nutrients through the acidification, eutrophication, and bioaccumulation of toxic substances [97,106,107]. Accumulation determines the bioaccumulation and transformation of pollutants in water. There is a link between the atmospheric deposition of PM and its effects on aquatic ecosystems. Monitoring is conducted using bioindicators, such as phytoplankton, organisms, species, or biological communities. Phytoplankton is the basis of the aquatic food chain, presenting diversity, and when the concentration of nutrients is high, its productivity can be altered with ecological consequences.

HMs in PM can be assimilated by the aquatic environment, regardless of whether they come from anthropogenic sources such as Cu, Fe, Al, Co, Pb, Mn, and Zn, volcanic eruptions, or vegetation fires. As, Cr, Se, and V result from fossil fuel combustion, vehicle emissions, and metal processing. HMs occur in waters in insoluble forms, which can subsequently become soluble or colloidal through metal–organic complexation processes [97,108]. As a rule, metals present in coarse PM have a low solubility compared to fine PM. The solubility of metals depends on the source and composition, the manner in which atmospheric deposition took place, whether it is wet or dry, the aging process of PM during long-distance transport, and even the tendency of PM to dissolve. The solubility of HMs in PM_2.5_ may be associated with the low pH of rainwater, which leads to the dissolution of metals before they reach the water surface, and these fractions become suitable for phytoplankton [109,110,111].

Atmospheric deposition of PM can be wet or dry. Wet deposition involves the natural capture of PM by water in the atmosphere and its deposition on the surface of soil and water, with each substance in the PM presenting different solubilities depending on the size of the PM. Dry deposition is a slower process that involves gravitational sedimentation for PM > 5 μm and turbulent diffusion or Brownian motion. For fine particles ranging from 0.2 to 2 μm, both processes are less efficient. These particles are carried over long distances from their source of occurrence and are deposited over time [34]. For instance, HMs were detected in the Martil basin area of Morocco, together with significant concentrations of As, Mn, and Fe, which can also occur through atmospheric deposition due to activities at ceramic units, marble factories, brickworks, and cement plants [112].

Singh et al. [75] assessed the air pollution tolerance index (APTI) and predicted the performance index (API) of *Dalbergia sissoo* Roxb. ex DC., *Mangifera indica* L., *Syzium cumini* (L.) Skeels, and *Psidium guajava* L. species to identify the most tolerant tree species and their efficiency in accumulating HMs and dust in their foliage near brick kilns. The highest APTI score was recorded by *M. indica* followed by *S. cumini*, *P. guajava*, and *D. sissoo*, with the scores decreasing as the distance from the brick kilns diminished. The high concentrations of Pb, Zn, and Cd found in *D. sissoo* indicated that this tree could serve as a bioindicator for these metals. Also, *D. sissoo* exhibited the highest metal accumulation index among all tree species studied.

Also, in marine waters, there are species of organisms sensitive to HMs, such as mollusks of the *Mytilius galloprovincialis* type, widespread in the Black Sea, being a bioindicator and sentinel for water quality, where Cd concentration is higher than European Communities’ limits (1 mg/kg) [113].

### 4.2. Soil

Soil is currently affected by numerous pollution phenomena that occur through climate change, emerging pollutants, and agriculture. Its remediation depends on the specifics of the area and the type of pollution. HMs are a result of anthropogenic activities, both at the soil and water level, but also occur through the deposition of various pollutants from the atmosphere.

In soil, the level of contamination varies depending on the purpose of the land. There is a link between plant variety and soil type, as well as study site (city) and the type of plant organ analyzed. Exposure to HMs in the urban environment is due to the poor air quality, emissions from vehicles on the roadside, the large number of pollution sources, and, in some cases, inadequate urban planning [114,115,116,117]. Some HMs such as Cu, Fe, Mn, Mo, Ni, and Zn have important physiological functions in soil. Their excess concentrations can cause toxic effects. Pb and Zn concentrations in roots and leaves of wild blackberry (*Rubus fruticosus* L. agg.) increased with their levels in the soil, while their translocation within this plant is diminished at higher soil levels [118].

These trends indicate the source of pollution more precisely. The presence of HMs in ecosystems leads to their consumption by the body, mainly through assimilation in the digestive system (food and drinks, especially milk, vegetables, and meat), skin contact, and the respiratory system (inhalation of exhaust gases) [119].

HMs indirectly affect the enzymatic activity of soil, such as Zn, or the microbial activity, reducing the pH or organic matter content, as in the case of Pb, Cd, and Hg. Other effects such as increased soil erosion may also be due to the presence of Ni or W, which may inhibit the role of soil microorganisms in the nutrient cycle. Effects such as reducing the number of *Azotobacter* sp. and actinomycetes may be due to the presence of Cr [120].

## 5. Health Effect

### 5.1. Heavy Metals

According to the State of the Global Air 2024 report, exposure to air pollution is linked to one in wight deaths worldwide [121]. The presence of PM in cities differs depending on population density, the activities carried out, and the geographical location, as there are known cities with low levels of PM. The abundance and composition of PM, including HMs, can vary depending on geographical location. Health effects are evident for certain population groups, including children, the elderly, pregnant women, and people with pre-existing respiratory or cardiovascular problems [5].

HMs can lead to toxic accumulation by forming stable and difficult to degrade compounds, especially in humans. This accumulation occurs through stable oxidation forms, such as Hg^2+^ or Cd^2+^, which react with biomolecules in the body, resulting in the formation of organo-metallic compounds. Certain metals play a vital role in the biological function of plants and animals, but their redox potential influences transport and homeostasis, as they can replace the original metal in protein sites, leading to cell damage [120]. There is a classification of HMs into essential (Cu, Ni, Fe, Zn) and non-essential (Cd, As, Hg, Pb) elements, but when concentrations are exceeded, toxicity occurs in any of the situations [122,123,124,125].

According to the International Agency for Research on Cancer (IARC), As, Cd, hexavalent chromium (Cr), and Ni are classified as Group 1 (carcinogenic to humans), Pb is classified as Group 2A (probably carcinogenic to humans), and many other metals are classified as Group 2B (possibly carcinogenic to humans) [60].

HMs enter the food chain and cause damage to the nervous system, circulatory system, kidneys, and liver. Penetration into the body through dermal absorption, inhalation, and ingestion routes can affect the nervous system and cause anemia when contaminated with Hg, As, and Pb. HMs affect and inhibit the mechanisms and enzymatic functions necessary for metabolism and interact with DNA and proteins, leading to carcinogenesis or apoptosis. HMs are mostly eliminated through urine and bile excretion in large quantities. Other organs of the body such as hair, nails, dan milk secretion and sweat also contribute to their elimination in smaller amounts [126].

Pb is neurotoxic, leading to attention-deficit hyperactivity disorder (ADHD) in children. Lead toxicity in humans is a public health issue that predominantly impacts children, with the primary effect of lead occurring in the brain [15,23,115].

The levels of As, Ni, and Pb detected in ambient air around Bakhtegan Lake (Iran) caused adults to suffer from higher cancer risks compared to children due to exposure to increased concentrations of As [60].

Cd is well known for its carcinogenic effects and poses risks to various organs of the body, including the lungs, kidneys, bones, prostate, and pancreas [126]. Cd is also associated with cardiovascular diseases, especially in smokers. It can enter the human body through the consumption of leafy vegetables, oilseeds, meat and organs, and nuts. It accumulates in the liver, leading to acute exposure that causes liver apoptosis and necrosis [23,115]. It was reported that Cd activates various types of diseases in humans, such as oxidative stress, DNA methylation, apoptosis, and DNA damage [127].

Cr induces mental and respiratory disorders, cancer, ulcers, and hyperkeratosis. The mechanism of toxicity and carcinogenicity of Cr consists of the intracellular reduction of Cr^6+^ to Cr^3+^. It has been observed that induced DNA lesions lead to the production of DNA adducts, chromosome aberrations, and DNA transcription [127].

Hg produces neurological damage in children, mental disorders, and damage to the digestive system. Hg can affect humans through a high consumption of marine and freshwater fish, affecting general cognitive ability. The literature indicates that about 15% of Hg is absorbed and about 80% penetrates through inhalation.

W is a carcinogenic agent which affects the respiratory tract of workers through inhalation and contributes to allergies and gastrointestinal discomfort.

Ni produces skin allergies, pulmonary fibrosis, and cardiovascular disease. Zn induces abdominal pain, nausea, vomiting, diarrhea, anemia, and irritability [120].

Pb influences growth in children and bone tissue lesions. Depending on the location and type of deposition, HM toxicity can lead to brain damage, cognitive function impairment, motor skill deficiency, memory disorders, and even dementia. It has been observed that in the plasma of patients suffering from Alzheimer’s, the levels of Hg, Cd, and Mn are increased [128].

Mn in small amounts acts as a neuroprotector, but prolonged exposure causes apoptotic cell death and Alzheimer’s. Cu and Zn can affect neurodevelopment and cause diseases such as schizophrenia and Wilson’s disease, although they are essential elements, being released in small amounts during synaptic firing [128]. In addition, epidemiological studies have indicated that water-insoluble HMs such as Zn cause an inflammatory response in the lungs [129].

### 5.2. Heavy Metals Combined with Particulate Matter

When HMs are associated with PM, they participate in oxidative reactions that induce oxidative stress. Metals such as Fe^2+^, Cu^+^, and Mn^3+^, as well as organic compounds such as quinones, produce reactive species of the O_2_⋅ and ^⋅^OH type in the presence of H_2_O_2_ through Fenton-type reactions [130].

**Figure 5:**
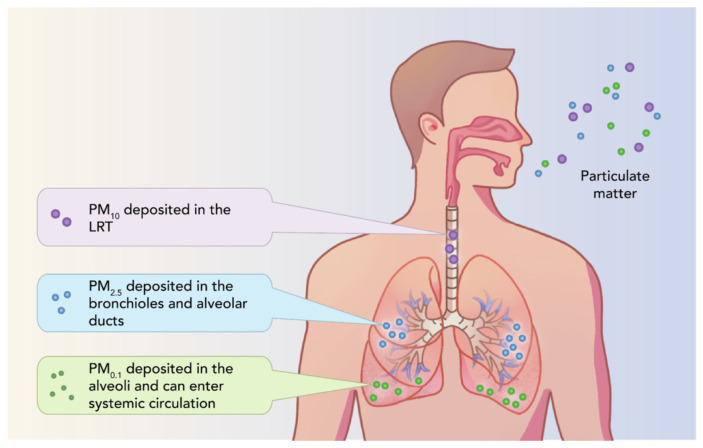
Deposition of inhaled PM in the body [101] (open access article distributed under the terms of the Creative Commons CC-BY license).

Inhaled PM reaches the pharynx and lower respiratory system, and through ingestion, the gastrointestinal system, leading to a series of pathologies. By inhalation into the trachea and bronchi, they can be partially eliminated via mucociliary actions. The location in the body depends on the size of the PM, as seen in Figure 5 [101].

Exposure to PM is associated with the risk of mortality due to respiratory diseases, such as chronic obstructive pulmonary disease (COPD) or acute lower respiratory tract infection and cardiovascular disease [131].

The WHO air quality guideline (AQG) established the annual average concentrations of PM_2.5_ up to 5 µg/m^3^ and up to 15 µg/m^3^ for 24 h average exposures for more than 3–4 days per year [132].

**Figure 6:**
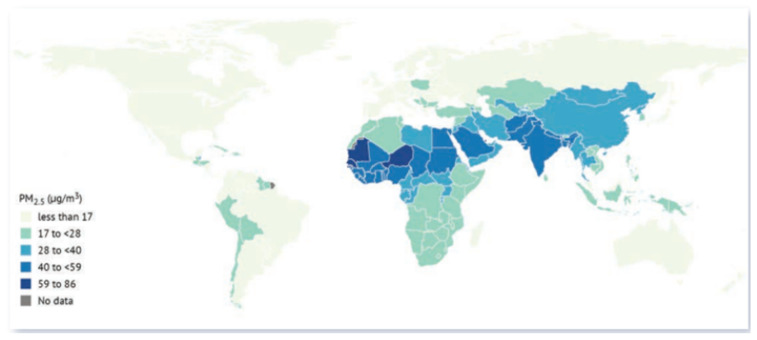
A worldwide map displaying the national population-weighted yearly average PM_2.5_ concentrations in 2020 [121] (open access).

Exposure to PM_2.5_ is divided into two categories: ambient air pollution (outdoor source) and household air pollution (indoor source) from the use of solid fuel for cooking. The latter affects over half of the world’s population, with particularly high levels in Asia and Africa [121]. Long-term exposure to PM_2.5_ compared to other air pollutants has resulted in poor health of those exposed, even in less polluted areas of Europe, Canada, and the United States, where levels do not exceed 4 µg/m^3^ [121]. Globally, PM_2.5_ concentrations are decreasing or starting to stabilize in some areas. The State of the Global Air 2024 report documented that the global average exposure to PM_2.5_ was 31.3 µg/m^3^ in 2020, with the highest levels recorded in South Asia, Africa, and Middle Eastern countries, as shown in Figure 6. The sources of PM_2.5_ differ by region [121].

The impact on human health also depends on the diameter of the particles. For example, the source of HMs in PM_10_ comes from soil and biological components. Large quantities of HMs and PAHs can be present in PM_2.5_, and in the case of PM_0.1_ nanoparticles, they can cross the biological barrier due to a large reactive surface, affecting internal organs.

Studies performed for PM_2.5_ monitoring have demonstrated harmful effects, leading to metabolic imbalances, various forms of cell death and dysfunction, genetic dysregulation, and exacerbation of cellular lesions. Although there are numerous studies in the direction of evaluating health status directly related to PM_2.5_, the effects of long-term exposure are still not elucidated [133].

For humans, inhalation is the most common route of entry for PM and HMs, followed by absorption through the skin and food consumption.

The skin responds to exposure to pollutants, especially PM_2.5_, through a series of reactions, leading to dermatitis. Damage to the skin barrier can result in immune system harm and inflammation. These effects can be reduced by consuming antioxidants and vitamin C [134].

The chemical composition of PM_2.5_ can undergo changes during transport through the atmosphere. In addition, it can exist alongside ultrafine PM_0.1,_ which is part of aerosols, for which there is evidence suggesting that these particles could reach the brain and lungs, causing direct damage. PM_0.1_ in aerosol form contains water-soluble compounds, mineral and organic carbon, and HMs.

Also, preclinical studies have established a link between PM exposure and ocular surface dysfunction, such as dryness, conjunctivitis, and allergies [135].

## 6. Biomonitors

PM pollution of plants is a major risk because once particles reach the surface of plants and enter tissues, they cannot be removed. Moreover, there are microorganisms that, when interacting with macroorganisms, lead to the occurrence of PM pollutants. Plant–microbe associations, although they can cause diseases and losses of symbiotic organisms, are also beneficial for the degradation of pollutants [99].

**Figure 7:**
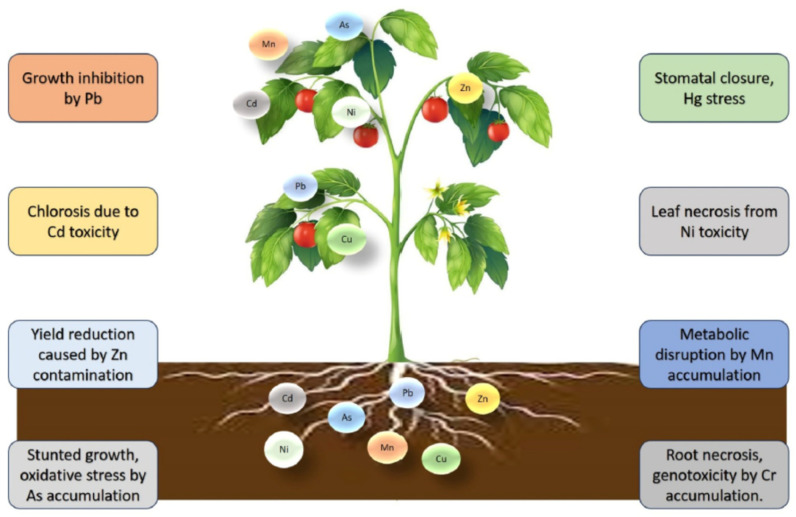
Impact of HMs on plant health. Reproduced from [120] with permission from Elsevier.

The tendency of HM accumulation in plants is illustrated in Figure 7 [120].

PM accumulation varies with concentration and distance from the emission source, meaning that in the same tree species, the accumulation levels could be different, with PM saturation being reached quickly in areas with high air pollution levels. Also, strong winds and heavy rains remove PM from the leaf surface, but a lighter rain favors the accumulation of PM on vegetation by adhering to “stickier” surfaces [136,137].

Forests play a key role in improving air quality and quality of life in urban areas through recreational benefits, supporting ecosystems through pollination and soil formation, and regulating them through flood control or the provision of water, fiber, etc. Gaseous pollutants are usually absorbed into the stomata and then into the intercellular spaces, while PM and HMs are either absorbed by tissues or remain on the leaf surface [138].

Plants are classified into three groups with regard to their uptake of HMs: metal excluders, indicators, and accumulators or hyperaccumulators. Excluders are those plants that prevent the translocation of HMs inside the plant, and the quantities of metals can be detected in the roots. HM indicators drive metals into aboveground tissues, and HM levels in tissues reflect metal levels in the soil. Hyperaccumulators are those plant species in which HMs are concentrated and migrate from the soil to aboveground tissues, exceeding the concentrations in the soil or in other nearby species. These types of plants are used in phytoremediation, having an obvious ecological role and preventing various diseases. There are species that accumulate a single metal [139].

The transfer of heavy metals from road traffic to plants may influence the activity of bees, which are also accumulators of HMs [140]. Medicinal plants can act as hyperaccumulators or excluders, an example being lavender, which due to its bioaccumulation capacity can play an important role in phytoremediation [141].

A short presentation of HM migration into soil and plants can be observed in Table 2.

The ecotoxicological hazards due to high levels of metals in the environment require continuous monitoring of the degree of contamination and the risk of exposure, both for plants and animals, which presents a danger to human health. One approach can be the use of organisms and plants as biological monitors (bioindicators) to establish the levels of metal pollution, such as fungi, lichens, tree bark, tree rings, vascular plants, and leaves of higher plants that have been used to detect the deposition, accumulation, and distribution of metals. For urban areas, higher plants are used, since lichens and mosses are often absent. The following species have been tested as biomonitors: *Pinus sylvestris*, *Picea abies*, and *Quercus ilex*.

The results presented in this review, however, took into account situations in which the concentrations in bioindicators were the highest, excluding, where not indicated, the effect of washing.

According to Markert and Wittig, a species that plays a role as a biomonitor has to be present in the analyzed area, be easy to identify and sample, and have the ability to differentiate metals as a source between air and soil [145,146].

The common dandelion (*Taraxacum officinale*) has been studied as a bioindicator among wild plants, being widespread, with a high tolerance to pollution, appearing in mining sites, on roads, and at metallurgical or thermal power plants. Studies have been carried out with this plant along highways in Poland, but also in contaminated urban areas in Europe, the USA, and Canada [115,147,148,149,150,151]. Dandelion has phytoextraction potential for polluted industrial areas. The accumulation of HMs is higher in roots than in leaves and depends on the distance and source of pollution, the type of metal, and the occurrence of morpho-structural changes, but also on the season in which the samples are taken [6,115].

### 6.1. Roots

HMs migrate into the soil through atmospheric deposition and chemical fertilizers. In close dependence, plants are influenced by the pollutant capture mechanism, usually having a system to reject HMs for metabolic functions. However, there are certain types of plants that have the potential for hyperaccumulation at ppm levels [120,152]. The capacity of a plant to absorb and concentrate metals from soil is expressed by the bioaccumulation factor (BAF), which is the ratio between the amount of HM in the plant tissue and that in the soil [153].

An excess or deficiency of HMs in plants can alter their biochemical and physicochemical functioning [120]. Metals such as Fe, Mo, Ni, Cu, and Zn are essential for plant growth and metabolism. On the other hand, toxicity in agricultural crops causes a major imbalance in the context of climate change.

Growth plants are influenced by weather conditions, climate, soil, and nutrients. Cd affects growth, decreases carotenoid and chlorophyll content, and leads to enzyme degradation. Zn leads to a decrease in photosynthesis, prevents leaf development, induces a low flower yield, and stops the absorption of Fe, Cu, and Mn as essential metals. Cr affects water balance and nutrient assimilation, induces changes in the plant cell, and causes chlorosis in leaves and a decrease in pigment levels, with the modification of various enzymatic functions.

The presence of Pb affects root length, chlorophyll and carotenoid content, and sugars, and causes turgor and necrosis. Ni causes inhibition of root branching, necrosis, and chlorosis. The presence of Hg decreases chlorophyll content, influences embryonic growth, seed germination, and water absorption. W decreases the growth rate of plants, causing structural abnormalities in cellular components.

Tripathi et al. studied the concentration of Bi, Co, Mo, Ga, and V in suspended particulate matter deposited on the roots of various plants in an urban area [143]. They found 38.66 ppm of Bi in the roots of *B. glabra*, 4.49 ppm of Co in *A. indica*, 14.06 ppm of Mo in the roots of *F. benjamina*, 50.35 ppm of Ga in *F. benjamina*, and 8.20 ppm of V in *P. longifolia*.

### 6.2. Leaves/Needles

Leaf tissue is commonly used as a bioindicator and bioaccumulator for the storage of pollutants in the atmosphere. The capacity of plants to accumulate metals has been known since the 16th century [154]. Factors such as season, plant age, soil type, temperature, and light must be taken into account in the study of bioaccumulation. Another important factor is leaf washing, which leads to a decrease in metal concentration in the plant during the exposure period.

Biomonitoring of plants, especially leaves, according to their characteristics, provides information on HM accumulation in urban areas. For example, Baldantoni et al. indicated that two mediterranean tree species are compatible with the assimilation of HMs and PAHs, holm-oak (*Quercus ilex* L.) and olive (*Olea europaea* L.), with the oak species being preferable for Cr, Fe, Mn, Ni, Pb, and V [15].

The washout dynamics for PM_1_, PM_2.5_, and PM_10_ show an exponential trend, similar to water-insoluble particles (0.2–100 mm) and water-soluble ions. Species such as *E. japonicus* show a high water retention capacity in leaves. Washout volumes were higher for coarse particles, and the proportion of large particles decreased and that of small particles increased with precipitation because small particles were trapped in the channels and subsequently removed after precipitation [155].

Urban plants play an essential role in PM capture, especially on leaf surfaces, and in the absorption of ultrafine PM into tissues through stomata [8]. Capture by deposition on leaf surfaces occurs through sedimentation for large PM, as well as derivative diffusion and surface contact and interception resulting from turbulent flow for fine and coarse PM. The wax layer can affect the interception and adhesion of PM, just as plant characteristics can influence deposition [43].

In the study of HM contamination, metal accumulation indices in plants and pollution tolerance indices are calculated. Nadgorska-Socha et al. indicated in their studies the tendency of some species, *Plantago lanceolata* < *Robinia pseudoacacia* < *Betula pendula* < *Taraxacum officinale*, to be used in urban areas with high soil and air contamination with heavy metals [6].

The capacities of plants to accumulate PM and PAHs varies based on species composition. In the study performed by Mukhopadhyay et al., eight plants from southeast Kolkata, India, namely *Nerium oleander*, *Tabernaemontana divaricata*, *Calotropis gigantea*, *Bauhinia acuminate*, *Polyalthia longifolia*, *Alstonia scholaris*, *Neolamarckia cadamba*, and *Plumeria alba*, were selected to examine their potential to act as vegetation barriers for the detection of PM and PAHs [156]. The authors showed that a concentration of PM ranging from 526.59 to 2731.76 μg/cm^2^ adhered to leaf surfaces. *T. divaricata*, *P. alba*, and *N. cadamba* were observed to accumulate 53–62% of PAHs.

Pine needles have been used in the monitoring of air pollutants, because their waxy surfaces accumulate gaseous pollutants and polluting particles. Aleppo pine (*Pinus halepensis* L.) was also tested as a biomonitor for Pb, Cd, Cu, and Zn pollution in the city of Amman, Jordan. Aleppo pines were preferred because they are available in large numbers in this city and easily accumulate heavy metals. Aleppo pine needles (*P. halepensis* L.) were found to be a good bioindicator for Pb, Cd, Zn, and Cu pollution in the city [23]. For example, the washing process for Aleppo pine needles (*P. halepensis* L.) significantly reduced Pb, Zn, Cu, and Cd concentrations, thus confirming the airborne deposition of these metals [23].

### 6.3. A Plant’s Influence on PM Concentration

The decrease in PM concentration in the atmosphere upon contact with plants is achieved through two mechanisms: deposition and dispersion.

The deposition mechanism can be dry or wet. Dry deposition is influenced by particle size in the absence of precipitation, and two phenomena can occur: diffusion for PM < 1 μm and gases, and deposition by contact with the leaf surface for PM_10_ and by sedimentation for PM > 10.

Wet deposition is influenced by meteorological phenomena, including high air humidity in the case of hygroscopic PM, which, through absorption, can accelerate the rate of deposition [138].

The dispersion phenomenon is diminished in the case of precipitation, which helps the deposition of dust on plants, just as winds make it difficult for PM to be deposited on the surface of leaves. Dispersion is also influenced by urban topography, the dilution that may occur due to winds, variations in airflow in buildings, and limitations regarding the morphology, height, and organization of plants. As a rule, evergreen plants, especially conifers, capture PM more easily due to their needles [138].

Temperature plays an important role in the deposition mechanism, especially since at high temperatures higher concentrations of PM occur due to biogenic VOC production, and the residence time in the atmosphere increases. Ultrafine PM more easily penetrates stomata that are much more open for water supply [136]. PM accumulation also depends on the leaves, crown features, and age of the trees. Also, the total surface area of the leaf is important for PM accumulation, but the amount accumulated on a tree is not directly proportional to that accumulated on the leaves of the trees [157]. Autumn leaf fall of deciduous trees leads to large seasonal variations related to PM accumulation. Also, broad elliptical and linear leaves adapt to the current created by wind and are less efficient in capturing PM than, for example, needles [158]. Leaves that have a coarse structure with grooves help capture particles more quickly [159]. A low stomatal density helps PM accumulation, but more stomata can also increase the thickness of the leaf surface, which favors PM deposition [160]. The amount of wax also helps PM deposition, especially for lipophilic organic pollutants.

## 7. Conclusions and Perspectives

Air pollution remains one of the top 15 biggest environmental problems of 2025, according to the Earh Organization, and it is closely linked with global warming, the most important threat [161].

The present review is focused on the characteristics of heavy metals (HMs) adsorbed by particulate matter (PM) from air, assessments of PM, sources of generating these pollutants in atmosphere, and the influence of HMs transported from air into the water and soil environment. The type of industry, modes of transport, and daily activities directly influence air quality and, consequently, the composition of dust and HMs.

This study revealed that the monitoring of HMs in PM is influenced by various factors, including industrial activities, traffic emissions, atmospheric conditions, and the proximity of sampling locations to pollution sources.

The actual tendencies in the preparation of samples containing HMs aim to enhance result quality, reduce the maintenance requirements of analytical equipment, and support the demand for environmentally sustainable procedures while ensuring the health and safety of personnel.

Increasing levels of HMs (Pb, Cd, As, Ni, Cr) in PM from presented sources lead to the development of real-time monitoring and AI-based prediction models for HM concentrations.

The accumulation of HMs through atmospheric deposition can lead to their buildup not only in soil and water but also in crops or plants, causing damage and alterations to physiological functions in animals or humans through the food chain.

Prolonged exposure to metals like As, Cd, and Pb can cause serious health problems, including respiratory issues, cardiovascular diseases, and cancers. These metals are of particular concern in urban and industrial areas where PM_2.5_ levels are high, as HMs have a preference to concentrate in PM_2.5_.

Accurate identification of pollutant types and concentrations is essential for establishing regulatory standards and developing long-term air quality strategies. Improving the biomonitoring of different part of plants for the assessment of HMs is highly recommended.

Rigorous control of emission sources, a transition away from fossil fuels, and investment in materials that enhance the properties of advanced air filtration systems are necessary actions for improving air quality. As the demand for PM measurement increases, there is a growing need for air filters that are not only highly effective but also renewable and biodegradable.

Future research on HMs adsorbed onto PM should focus on understanding their transfer from both natural and anthropogenic sources, exploring the mechanisms of adsorption, and investigating innovative materials for their efficient removal. A thorough assessment of the long-term environmental and health impacts of these contaminants is also essential. Additionally, the presence of other pollutants such as NOx, CO_2_, SO_2_, VOCs, PAHs, and O_3_ bound in PM should be studied to better understand their environmental risks and potential effects on human health. Future studies should also involve a larger number of samples to gain more comprehensive insights into the health risks posed to humans.

Particulate matter (PM) is the air pollutant driving the most important health issues. Even though there are many techniques for monitoring and analytical detection, there is an identified gap in policy and regulations and knowledge of health effects, so new approaches are needed to create effective solutions in line with scientific progress as well as other environmental problems.

Because of the associated health problems, more epidemiological studies regarding the link between HM exposure and respiratory, cardiovascular, and neurological diseases will be undertaken in the next few years. Also, research on the risks of long-term bioaccumulation in soil, water, and food chains will establish the accumulation mechanisms and provide green infrastructure solutions for capturing HMs and implicitly PM.

Current trends in air pollution related to PM and HMs highlight the growing concerns about ultrafine PM and non-exhaust sources, while advancements in monitoring, stricter regulations, and green solutions, such as vegetation-based urban infrastructure, are becoming increasingly relevant for mitigating their impact on human health and the environment.

## Figures and Tables

**Table 1:** Filter properties used for PM extraction from air (adapted from [65]).

No. crt.	Filter Material	Efficiency %	Other Characteristics	Reference
1	PAN nanofiber on window mesh	92.6% for PM_10_ 91.2% for PM_10_ and PM_2.5_ 90.6% for PM_2.5_	PM_2.5_ mass concentration >708 μg/m^3^	[66]
2	PLA with high molecular weight	92.6% for PM_0.3_	630 nm size	[67]
3	PAN nanofiber filters Ti_3_C_2_Tx MXene nanosheets	95% for PM_1_, PM_2.5_, and PM_10_	Low pressure drop of ∼42 Pa MXene nanosheets strongly inhibit the propagation of bacteria (e.g., *E. coli* and *S. aureus*) in the filters	[68]
4	Zein fibers	99% (PM_0.3_)	Lower pressure drop (109 Pa)	[69]
5	Nonwoven filter fibers activated with carbon black	32.9–46.0% for PM_10_ 18.7–30.8% for PM_2.5_ 12.6–24.2% for PM_1.0_	Filtration velocity of 0.8 m/s	[70]

**Table 2:** Level of HMs detected in soil and different parts of plants.

No. Crt.	HM Type	Soil, mg/kg	Roots, mg/kg	Leaves/Needles, mg/kg	Plants	Ref.
1	Cd industry area; Roadside; Suburban area	10 3.2 2.1		3.5 2.20 0.55	Aleppo pine (*Pinus halepensis* L.)	[23]
	Cd	0.4–3.6	0.4–2.8	0.5–3.9	*Taraxacum* *officinale*	[115]
		0.27		0.10	*Inula viscosa* L.	[142]
		2.80		1.29 1.55 0.72 0.65	*T. officinale* *P. lanceolata* *B. pendula* *R. pseudoacacia*	[142]
			2.65		*F. religiosa*	[143]
2	Cr	23.2–40.6	14.0–26.1	15.8–24.8	*Taraxacum* *officinale*	[115]
3	Ni	2.1–13.2	0.2–42.1	0–3.9		[115]
			65.40		*B. glabra*	[143]
		18.9		4.87	*Inula viscosa* L.	[142]
4	Pb	27.0–231.5	4.3–34.2	3.0–9.5	*Taraxacum* *officinale*	[115]
			12–19		*Citrus limon* (L.)	[144]
		98		28.07 25.38 16.02 11.08	*Taraxacum officinale* *Plantago lanceolata*, *Betula pendula* *Robinia pseudoacacia*	[6]
		87.4		7.25	*Inula viscosa* L.	[142]
			63.18		*F. benghalensis*	[143]
	Pb industry area; Roadside; Suburban area	160 500 80		90 196 40	Aleppo pine (*Pinus halepensis* L.)	[23]
5	Zn industry area; Roadside; Suburban area	1210 215 115		262 95 55	Aleppo pine (*Pinus halepensis* L.)	[23]
	Zn		46		*Citrus limon* (L.)	[144]
		82.2		47.6	*Inula viscosa* L.	[142]
		550.1		179.82 97.62 389.05 57.72	*Taraxacum officinale* *Plantago lanceolata*, *Betula pendula* *Robinia pseudoacacia*	[6]
			460.13		*F. religiosa*	[143]
6	Cu industry area; Roadside; Suburban area	40 85 18		29.5 37 16.5	Aleppo pine (*Pinus halepensis* L.)	[23]
	Cu		12		*Citrus limon* (L.)	[144]
		60.4		10.6	*Inula viscosa* L.	[142]
		14.65		10.09 5.85 2.94 1.78	*Taraxacum officinale* *Plantago lanceolata*, *Betula pendula* *Robinia pseudoacacia*	[6]
			391.02		*P. longifolia*	[143]
7	Mn		20 to 56		*Citrus limon* (L.)	[144]
		224		140	*Inula viscosa* L.	[142]
		244.3		24.76 47.12 63.27 25.18	*Taraxacum officinale* *Plantago lanceolata*, *Betula pendula* *Robinia pseudoacacia*	[6]
			163.82		*F. religiosa*	[143]
8	Fe	15,700		730	*Inula viscosa* L.	[142]
			4800.81		*F. benghalensis*	[143]
		1021.3		159.18 299.05 260.02 192.11	*Taraxacum officinale* *Plantago lanceolata*, *Betula pendula* *Robinia pseudoacacia*	[6]
9	Cr	42.4		7.03	*Inula viscosa L*.	[142]
			358.27		*P. longifolia*	[143]

## Data Availability

The data presented in this study are available on request from the corresponding authors.
